# The Physics of Steam Sterilisation

**Published:** 1900-06-09

**Authors:** 


### THE PHYSICS OF STEAM STERILISATION.

In regard to the question raised by the above story in regard to the reliability of steam sterilisation as usually practised, attention may be drawn to some experiments by Dr. Dunham[1] of New York, which go to prove that if the steam in a steriliser is to be brought into efficient contact with the articles to be sterilised provision must be made for the escape of the air contained in the apparatus, and that this escape should by preference take place at the bottom. He points out that experiments have fully proved that boiling water or saturated steam at the atmospheric pressure will surely kill all pathogenic micro-organisms in the course of a very few minutes, and that the practical problem in sterilising is how to bring the boiling water or the suturated steam into contact with all parts of the objects to be submitted to its influence. This is a question of physics, and in solving it one must bear in mind that steam has a much lower specific gravity than air, the ratio being about as six is to ten, and that air is a very poor conductor of heat. Thus it happens that unless the air contained in the apparatus is got rid of it lies at the bottom, where it remains practically unaffected by the steam above. Hence the necessity, if it is desired that the full temperature shall be reached in every part of the apparatus in a reasonable time, that the steam should come in at the top, and should blow the air out before it at the bottom. The experiments were made in a cylinder six inches in diameter and nearly three feet high, through the sides of which four thermometers projected—one near the top, another near the bottom, and the other two at equal intervals between them, the time which it took each of these thermometers to rise to 98 deg. Cent., or to the highest point to which it ever did rise, being noted. The difference of time, according to the positions of the inlet and the outlet, were very marked, the best results being obtained when the steam entered at the top and blew out at the bottom, and far away the worst when there was only one opening, and that one at the top. In this case the temperature at the bottom never rose anywhere near the boiling point, although the experiment was continued for a long time. In another series in which the cylinder was filled with cotton, and thus represented more accurately the conditions existing in ordinary practice, the direction of the current of steam, so long as there was a current, was found to exert much less influence. In fact, it would appear that when the cylinder was stuffed with cotton, so that there was no internal circulation, the steam passed from end to end in a compact column, and it did not much matter which way it went. The same lack of circulation, however, had a disastrous effect when the attempt was made to do with only one opening; for whether that was placed at the top or at the bottom, the steam failed to raise the temperature evenly; and in the case where the opening was at the top alone there was a complete failure to do more than heat the very uppermost layers, the two lower thermometers remaining unmoved, notwithstanding that the one at the top was almost at a boiling temperature. The moral of these experiments, and of others dealing with high pressure sterilisers fitted with "ejectors," is that in whatever form steam is used, unless there is a complete blow through, there is no security that the heat will reach every part of the contents.

[1] The Post Graduate, May, 1900.

